# PBMC-derived FGF, PDGF, VEGF and GM-CSF secretion in endometriosis: a case–control *in vitro* study

**DOI:** 10.3389/fmed.2026.1821695

**Published:** 2026-06-04

**Authors:** Marcin Sadlocha, Aleksandra Krzywon, Jakub Marcin Staniczek, Jakub Toczek, Zenon Czuba, Rafal Stojko

**Affiliations:** 1 Chair and Clinical Department of Gynaecology, Obstetrics and Gynaecologic Oncology, Faculty of Health Sciences, Medical University of Silesia in Katowice, Katowice, Poland; 2 Department of Applied Informatics, Silesian University of Technology, Gliwice, Poland; 3 Department of Biostatistics and Bioinformatics, Maria Sklodowska–Curie National Research, Institute of Oncology, Gliwice Branch, Gliwice, Poland; 4 Department of Microbiology and Immunology, Faculty of Medical Sciences in Zabrze, Medical University of Silesia in Katowice, Katowice, Poland

**Keywords:** angiogenesis, cytokines, endometriosis, growth factors, *in vitro* culture, peripheral blood mononuclear cells

## Abstract

**Background:**

Endometriosis is a chronic inflammatory disease with immune dysregulation in which angiogenic, and hematopoietic mediators are thought to contribute to ectopic lesion establishment and persistence. Whether circulating immune cells are intrinsically primed to secrete higher levels of pro-angiogenic growth factors remains unclear. This study evaluated *in vitro* secretion of fibroblast growth factor (FGF), platelet-derived growth factor (PDGF), vascular endothelial growth factor (VEGF) and granulocyte–macrophage colony-stimulating factor (GM-CSF) by peripheral blood mononuclear cells (PBMCs) from women with and without endometriosis.

**Methods:**

In a case–control design, women with laparoscopically and histopathologically confirmed endometriosis (*n* = 36) and laparoscopically confirmed controls without endometriosis (*n* = 44) were enrolled. PBMCs were isolated from peripheral blood and cultured for 24 h under basal conditions or after non-specific mitogenic activation with phytohemagglutinin (PHA, 5 μg/mL). Supernatant concentrations of FGF, PDGF, VEGF and GM-CSF were quantified using a multiplex bead-based immunoassay (Bio-Plex/Luminex). Between-group comparisons used nonparametric tests; univariate logistic regression explored associations with endometriosis status; and false discovery rate (Benjamini–Hochberg) adjustment was applied for multiple testing.

**Results:**

Baseline secretion of FGF, PDGF, VEGF and GM-CSF by PBMCs did not differ significantly between women with endometriosis and controls after correction for multiple comparisons. PHA stimulation induced marked shifts in secretion profiles across participants— characterized by increases in FGF, PDGF and VEGF and a decrease in GM-CSF—but neither stimulated concentrations nor percent changes differed significantly between groups following false discovery rate adjustment. In univariate logistic regression analyses, none of the baseline growth-factor measures significantly predicted the presence of endometriosis.

**Conclusion:**

Under standardized *in vitro* culture conditions, PBMCs from women with endometriosis do not show a generalized increase in secretory capacity for the evaluated pro-angiogenic/hematopoietic growth factors compared with PBMCs from controls without endometriosis. These data do not support systemic PBMC hypersecretion of FGF, PDGF, VEGF and GM-CSF in endometriosis and are consistent with a compartmentalized model in which disease-relevant pro-angiogenic signaling is predominantly shaped within local peritoneal and lesion microenvironments.

## 1 Introduction

Endometriosis is a chronic, estrogen-dependent gynecological disease affecting approximately 10% of women of reproductive age worldwide (1). It is characterized by the growth of endometrial-like tissue outside the uterine cavity—most commonly on the pelvic peritoneum, ovaries, and rectovaginal septum—leading to chronic pelvic pain, dysmenorrhea, dyspareunia, and subfertility (2–4). Despite substantial progress in clinical management, no definitive cure exists; hormonal therapy and surgical excision provide mainly symptomatic relief and recurrence remains common (4, 5). These limitations underscore the need to better define the mechanisms that enable lesion persistence, with increasing emphasis on immune dysregulation and the balance between inflammation and immune tolerance (1, 6).

The most widely accepted pathogenic framework, originally proposed by Sampson, posits that retrograde menstruation transports viable endometrial cells into the peritoneal cavity, where they adhere, implant, and proliferate (3, 4). However, retrograde menstruation is common, whereas clinically significant endometriosis develops only in a subset of individuals (3). This discrepancy suggests that additional determinants—particularly immunological, genetic, and hormonal factors—shape susceptibility to lesion establishment and progression (1, 3, 7). Once established, ectopic tissue survival depends on both evasion of immune-mediated clearance and the formation of new blood vessels (angiogenesis), processes supported by a dysregulated cytokine and growth factor milieu (2, 8).

Angiogenesis is crucial for the maintenance of endometriotic lesions, providing oxygen and nutrient delivery to ectopic tissue (2, 8). The lesion and peritoneal microenvironment are considered pro-angiogenic, with vascular endothelial growth factor (VEGF) frequently implicated as a central mediator; peritoneal fluid in endometriosis has been reported to contain higher VEGF levels than that of controls (2, 9). Other growth factors, including fibroblast growth factor (FGF/bFGF) and platelet-derived growth factor (PDGF), have also been described as elevated locally and linked to stromal cell proliferation, inflammation, and tissue remodeling (8, 10, 11). Granulocyte–macrophage colony-stimulating factor (GM-CSF) further connects inflammation with vascular and myeloid pathways and has been reported to increase in endometriosis in several studies, although results vary depending on compartment, disease stage, and detection method (10, 12, 13). Collectively, these data support a model in which the local lesion/peritoneal niche is enriched in pro-angiogenic and immune-modulating mediators (10, 12).

Immune dysregulation in endometriosis is not confined to the peritoneal cavity and may also be reflected systemically. Altered activation and cytokine production have been reported in circulating immune cells, including peripheral blood monocytes and granulocytes, suggesting that chronic inflammatory signaling may extend beyond local lesions (1, 14). Recent high-dimensional immune-profiling studies further support this concept. Mass cytometry analysis has demonstrated a distinct immune environment in the peritoneal fluid of women with endometriosis, while deep immunophenotyping studies have reported dysregulation of the mononuclear phagocytic system in both endometrial tissue and peripheral blood (15, 16). These findings suggest that systemic immune alterations may coexist with compartment-specific immune remodeling within the peritoneal and lesion microenvironment. Differences in circulating regulatory T cells (Tregs) have also been described, with some studies reporting higher peripheral frequencies (17) and others suggesting redistribution toward lesion sites, consistent with immune cell trafficking between blood and the peritoneal compartment (18). In parallel, endometriotic lesions may secrete chemokines such as CCL17, CCL22, and CCL20 that contribute to recruitment of immunoregulatory populations and shaping of a permissive immune environment (18, 19). Importantly, these observations emphasize systemic immune involvement while highlighting that immunoregulatory effects may operate through complex, compartment-specific networks rather than a single cell type or mediator (6, 20, 21).

Against this background, the key unresolved question is not whether angiogenic mediators are elevated locally, but whether circulating PBMCs exhibit an intrinsically altered ex vivo secretory phenotype in endometriosis. While extensive work has examined cytokine and growth factor concentrations in serum and peritoneal fluid, comparatively few studies have directly evaluated the intrinsic *in vitro* secretory capacity of peripheral blood mononuclear cells (PBMCs) with respect to key angiogenic and hematopoietic growth factors. PBMC culture offers a standardized approach to assess whether circulating immune cells from women with endometriosis display altered baseline secretion and/or heightened responsiveness following mitogen stimulation—features that could reflect systemic immune conditioning (1, 14).

To address this question, we compared basal and PHA-stimulated secretion of FGF, PDGF, VEGF, and GM-CSF by PBMCs from women with surgically confirmed endometriosis and laparoscopic surgical controls without visible endometriosis. Because the assay used unfractionated PBMCs and a single 24-h timepoint, all mechanistic and subgroup inferences were considered exploratory. PBMCs were cultured under basal and phytohemagglutinin (PHA)-stimulated conditions and growth factor concentrations were quantified using a multiplex bead-based immunoassay (Bio-Plex® system). We additionally performed subgroup analyses according to endometriosis stage (rASRM I–II vs. III–IV) to explore whether disease severity influences PBMC-derived growth factor profiles. We hypothesized that PBMCs from women with endometriosis exhibit an altered secretory phenotype, reflected by differences in growth factor release under basal conditions and/or after stimulation. Notably, our study does not test therapeutic targeting; therefore, any translational implications remain hypothesis-generating.

## 2 Materials and methods

### 2.1 Study design and participants

Recruitment took place between 2017 and 2019 at the Chair and Clinical Department of Gynecology, Obstetrics and Gynaecologic Oncology, Medical University of Silesia, Poland, as part of a single-center case–control project. We conducted a case–control study involving two groups of women of reproductive age (18–53 years), presumed to have reproductive potential unless clinically contraindicated. The endometriosis group consisted of 36 patients diagnosed with endometriosis via laparoscopic surgery. Diagnosis was confirmed by direct visualization of lesions and histopathology, and disease stage was recorded according to the revised American Society for Reproductive Medicine (ASRM) criteria (stages I–IV). These patients are those undergoing laparoscopy for chronic pelvic pain, infertility, or ovarian endometrioma resection. Controls were recruited from the same source population, namely women undergoing laparoscopy for infertility and/or pelvic pain in whom no endometriosis was visualized intraoperatively. We use the term surgical controls because alternative gynecologic conditions may have been present in this group. All participants were between 18 and 53 years old, and all provided informed consent before enrolment. The study protocol was approved by the Institutional Ethics Committee (No. KNW/0022/KB1/100/17; October 3rd 2017) in accordance with the Declaration of Helsinki, and informed consent was obtained from each subject prior to sample collection (Table 1).

**Table 1:** Inclusion and exclusion criteria.

Inclusion criteria	Exclusion criteria
Women aged 18–53 years, premenopausal and not pregnant, scheduled for diagnostic or operative laparoscopy. For the endometriosis group: laparoscopic confirmation of endometriotic lesions, irrespective of disease stage. For the control group: no visible endometriosis at laparoscopy. Written informed consent provided before enrolment.	Refusal or inability to provide informed consent. Use of immunosuppressive or hormone-altering drugs within the previous 3 months. Active acute infection or inflammation at the time of surgery. Active autoimmune disorder, chronic inflammatory disease, or any systemic disease that could affect immune parameters. Pregnancy. History of malignancy.

Control participants were surgical controls undergoing laparoscopy for infertility and/or pelvic pain, in whom no visible endometriosis was detected intraoperatively. They should therefore not be interpreted as healthy asymptomatic controls.

### 2.2 Sample collection

At the time of laparoscopy, peripheral blood samples (10 mL) were collected into heparinized tubes by antecubital venipuncture between 7:00 and 9:00 a.m. to minimize diurnal variation in immune parameters. For women with regular menstrual cycles, laparoscopies were preferentially scheduled between cycle days 5 and 12, based on calendar counting from the first day of the last menstrual period, to sample participants in the early–mid follicular phase. In women with irregular cycles, blood sampling was performed within this window whenever clinically feasible, but precise phase assignment could not be confirmed biochemically.

### 2.3 PBMCs isolation, culture and stimulation

PBMCs were resuspended in complete RPMI 1640 medium (10% heat-inactivated fetal bovine serum, 2 mM L-glutamine, 100 U/mL penicillin, 100 μg/mL streptomycin) at a density of 1 × 10^6^ viable cells/mL, as determined by trypan blue exclusion (viability ≥ 95% in all preparations). Cells were seeded in 24-well flat-bottom plates at 1 mL per well (1 × 10^6^ cells/well). For each participant, two wells served as unstimulated controls and two were stimulated with phytohemagglutinin (PHA-L, Sigma-Aldrich) at 5 μg/mL. Cultures were incubated for exactly 24 h at 37 °C in a humidified 5% CO₂ atmosphere, after which supernatants were collected by centrifugation (300 × g, 5 min, 4 °C), aliquoted and stored at −80 °C until analysis. By comparing stimulated and unstimulated conditions, we assessed both baseline growth-factor secretion by PBMCs and their capacity for altered secretion after non-specific mitogenic stimulation. Importantly, performing identical cultures for both endometriosis patients and controls under the same conditions allowed a direct comparison of the intrinsic differences in PBMC secretory profiles between endometriosis patients and controls.

### 2.4 Cytokine and growth factor measurement

The concentrations of the four target cytokines/growth factors – FGF, PDGF, VEGF, and GM-CSF – were measured in the culture supernatants using a multiplex bead-based immunoassay (Bio-Plex Pro Human Cytokine 27-plex Assay #M500KCAF0Y, Bio-Rad Laboratories). This technology is based on Luminex xMAP, which uses fluorescently color-coded microspheres coated with capture antibodies specific to each analyte. In our assay, a “sandwich” immunoassay format was employed for each target: antibody-coated beads bind the cytokine of interest, and a detection antibody labeled with a reporter (fluorophore) bind to the captured cytokine. By using beads of different colors for each cytokine, all analytes can be measured simultaneously in a small volume of sample. We utilized a commercially available multiplex kit with appropriate sensitivity (typically in the pg./mL range). The assay was performed according to the manufacturer’s instructions. Briefly, thawed supernatant samples (and known-standard calibration samples for each cytokine) were incubated with the antibody-coated beads in 96-well filter plates. After washing, detection antibodies and streptavidin-PE (phycoerythrin) were added to generate a fluorescent signal. The plate was read on the Bio-Plex analyzer, which detects the bead identity and quantifies the bound reporter signal, translating into concentration values based on the standard curves. Each sample was run in duplicate to ensure accuracy, and quality controls were included.

### 2.5 Data collection and outcome measures

For each participant, we obtained the concentration (in pg./mL) of each growth factor under both unstimulated and PHA-stimulated conditions. The primary outcomes were the concentrations of FGF, PDGF, VEGF, and GM-CSF secreted by cultured PBMCs. These were compared between the endometriosis group and the control group. We also noted each patient’s clinical data (age, BMI, endometriosis stage, etc.) to explore any correlations or confounders. Since our hypothesis focuses on differences between patients and controls, the key comparisons were: (a) baseline (unstimulated) secretion in endometriosis vs. controls, and (b) PHA-stimulated secretion in endometriosis vs. controls, for each of the factors. Additionally, the fold-change upon stimulation (stimulated vs. baseline for everyone) can be compared between groups to see if endometriosis PBMCs have an exaggerated or blunted response relative to PBMCs from surgical controls without visible endometriosis.

### 2.6 Statistical analysis

No formal *a priori* sample-size calculation was performed because the study was based on consecutive recruitment of eligible participants during the predefined recruitment period. The final sample included 36 women with laparoscopically and histopathologically confirmed endometriosis and 44 laparoscopic surgical controls without visible endometriosis. To provide a statistical justification for the available sample size, a *post hoc* sensitivity calculation was performed for a two-sided comparison between two independent groups, assuming *α* = 0.05 and 80% power. With the achieved group sizes (n = 36 and n = 44), the study had sufficient power to detect approximately moderate-to-large between-group differences, corresponding to a standardized effect size of about Cohen’s d = 0.64. Therefore, this study should be considered exploratory and powered primarily to detect moderate-to-large between-group differences; smaller effects cannot be excluded. Duplicate wells were averaged at the participant level before inferential analyses. Values below the lower limit of quantification were assigned LLOQ/2. Values above the upper limit of quantification were assigned the upper limit of quantification. Missing data were not imputed.

Categorical variables were described using counts and percentages, while continuous variables were expressed as medians with the first and third quartiles. For between-group comparisons, unstimulated and stimulated concentrations were analyzed separately using the Wilcoxon rank-sum test. Within-participant changes after PHA stimulation were assessed using the Wilcoxon signed-rank test. Percent change was calculated as 100 × (stimulated − baseline)/baseline; because baseline values close to zero can inflate percentage-based metrics, absolute differences and log2 fold change were also examined in Supplementary materials. For logistic regression, continuous predictors were scaled per 100 pg./mL or per 1 SD to improve interpretability of odds ratios. All subgroup and regression analyses were considered exploratory. Benjamini–Hochberg false discovery rate correction was applied within each family of related comparisons. A two-tailed *p*-value of less than 0.05 was considered statistically significant. All analyses were conducted using R statistical software, version 4.0.1.

The methodology outlined above ensures a controlled comparison of PBMC/lymphocyte function. By culturing cells *in vitro*, we aim to isolate the intrinsic differences in cytokine secretion capacity, minimizing external influences (all samples are treated identically in the assay). This approach, combined with robust patient selection criteria, addresses the research question in a systematic manner.

## 3 Results

This section summarizes the clinical characteristics of the study population and the *in vitro* secretion profiles of FGF, PDGF, VEGF and GM-CSF by PBMCs in women with and without endometriosis.

### 3.1 Demographic and clinical characteristics

A total of 80 women were enrolled: 36 with laparoscopically and histopathologically confirmed endometriosis and 44 controls without endometriosis confirmed at laparoscopy. Baseline demographic and clinical characteristics were comparable between groups (Table 2). There were no significant differences in age, anthropometric measures (body mass, height, BMI), obstetric history, or menstrual cycle regularity (all *p* > 0.39), indicating that the groups were well matched with respect to measured potential confounders.

**Table 2:** Demographic and clinical characteristics of women with endometriosis (+) and controls without endometriosis (−).

Characteristic	Surgical controls without visible endometriosis (*n* = 44)	Endometriosis (*n* = 36)	*p* value
Age, years	37.5 (30.8, 42.0)	37.5 (32.0, 41.0)	0.57
Body mass, kg	62.0 (56.5, 72.0)	62.0 (56.3, 80.0)	0.44
Height, cm	164.5 (160.0, 170.3)	165.0 (162.0, 168.3)	0.53
BMI, kg/m^2^	22.8 (19.8, 26.6)	22.8 (20.2, 29.0)	0.39
Pregnancies, *n* (%)			0.65
0	13 (29.5%)	16 (44.4%)	
1	18 (40.9%)	11 (30.6%)	
2	8 (18.2%)	6 (16.7%)	
3	4 (9.1%)	3 (8.3%)	
5	1 (2.3%)	0 (0.0%)	
Menstrual bleeding pattern, *n* (%)			0.54
Irregular	24 (57.1%)	16 (50.0%)	
Regular	18 (42.9%)	16 (50.0%)	

Continuous variables are presented as median (Q1, Q3); categorical variables as *n* (%). Percentages are presented with one decimal place and may not sum to 100.0 because of rounding. Comparisons were performed using the Wilcoxon rank-sum test (continuous) and Fisher’s exact or Pearson’s Chi-squared test (categorical).

### 3.2 Baseline growth factor secretion by cultured PBMCs

**Figure 1:**
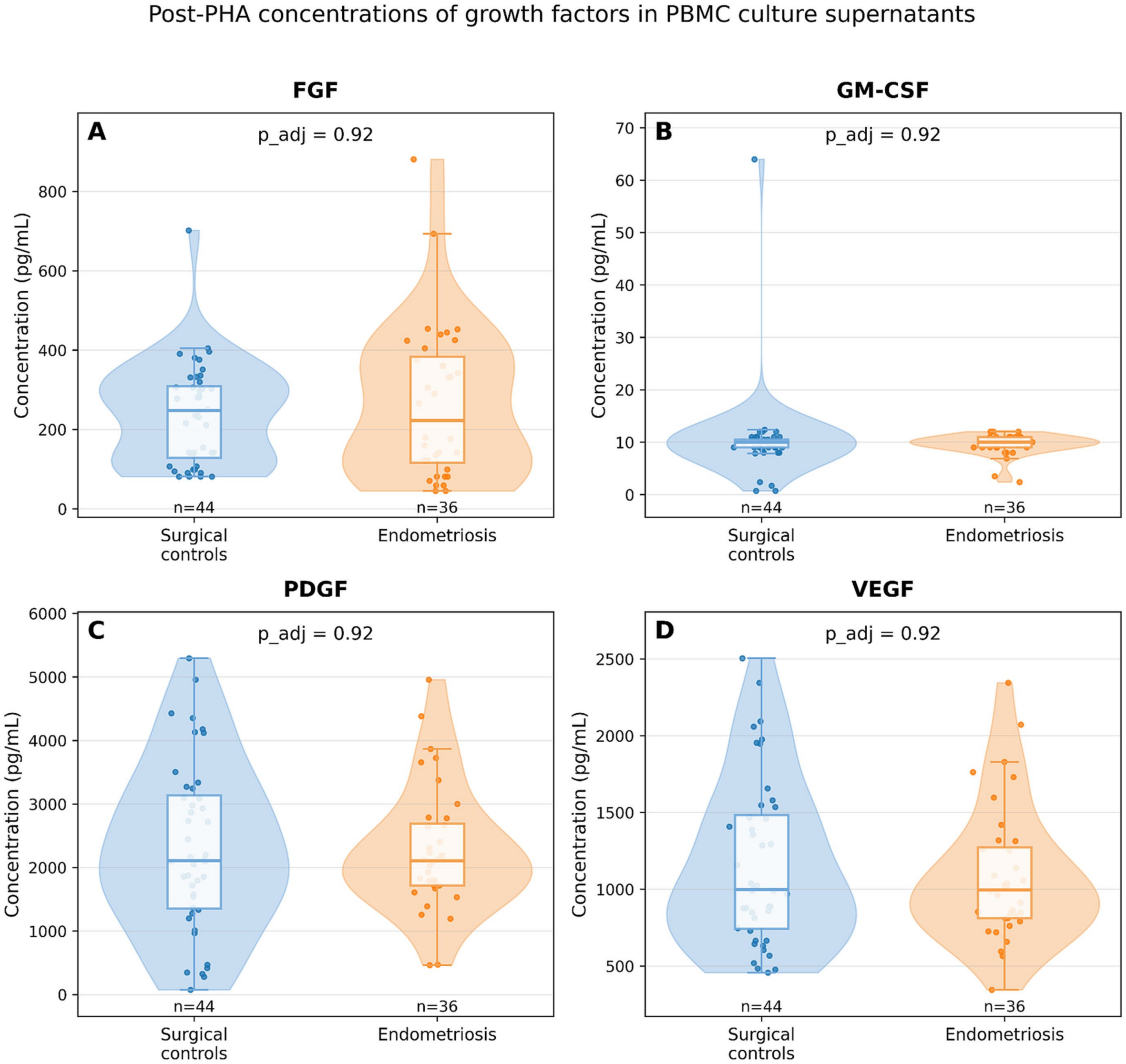
Baseline concentrations of FGF **(A)**, GM-CSF **(B)**, PDGF **(C)**, and VEGF **(D)** in PBMC culture supernatants from surgical controls without visible endometriosis (−) and women with endometriosis (+). Violin plots display individual-value distributions, embedded boxplots show medians and interquartile ranges, and dots represent individual participants. Concentrations are expressed in pg/mL.

Baseline (unstimulated) concentrations of FGF, PDGF, VEGF, and GM-CSF in PBMC culture supernatants did not differ between the endometriosis and control groups (Table 3; Figure 1). After Benjamini–Hochberg correction for multiple testing, none of the between-group comparisons reached statistical significance (all p-adj ≥ 0.76). Median FGF levels were identical in both groups (69.9 pg./mL), while PDGF and GM-CSF showed numerically lower medians in endometriosis that did not remain significant after correction (Table 3).

**Table 3:** Baseline (pre-stimulation) growth factor concentrations (pg/mL) in PBMC culture supernatants.

Characteristic	Surgical controls without endometriosis (−), *N* = 44	Endometriosis (+), *N* = 36	*p*-value	*p*-adj^1^
FGF 0*, pg/mL	69.9 (52.7, 83.9)	69.9 (52.7, 83.9)	0.42	0.84
GM-CSF 0*, pg/mL	32.4 (4.9, 51.5)	23.3 (4.4, 73.1)	0.76	0.98
PDGF 0*, pg/mL	807.7 (528.8, 1,104.8)	631.0 (381.1, 1,023.6)	0.19	0.76
VEGF 0*, pg/mL	761.5 (321.4, 852.2)	680.7 (396.8, 853.6)	0.98	0.98

Values are presented as median (Q1, Q3). ^1^ Adjusted p-values were calculated using the Benjamini–Hochberg procedure. 0* — before PHA stimulation. Patients with endometriosis are marked as (+), and surgical controls without endometriosis as (−). Data are presented as median (Q1, Q3). *p*-values were calculated using the Wilcoxon rank-sum test; adjusted *p*-values (p-adj) were derived using the Benjamini–Hochberg procedure.

### 3.3 Growth factor response to PHA stimulation

**Figure 2:**
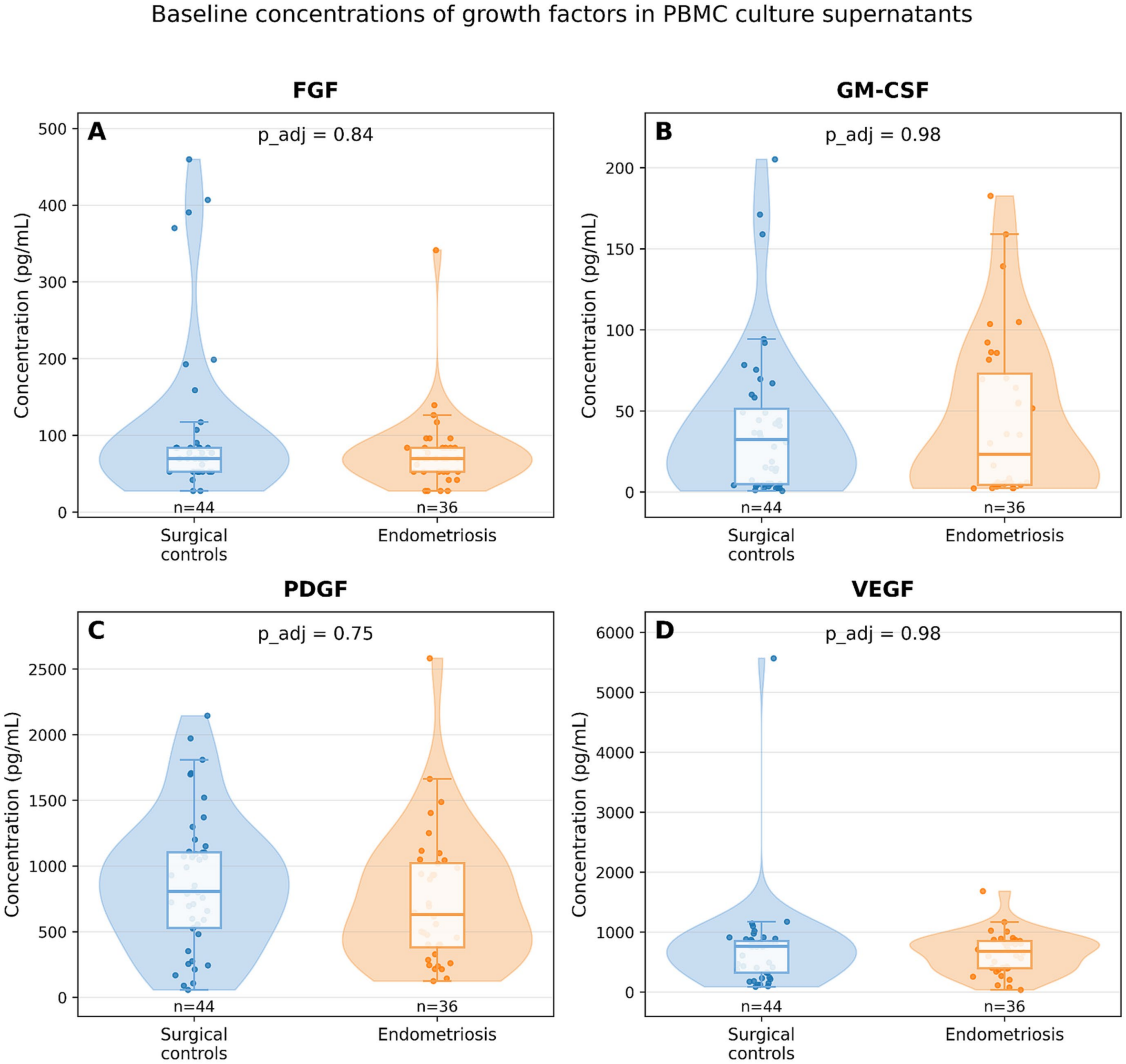
Post-PHA-stimulated concentrations of FGF **(A)**, GM-CSF **(B)**, PDGF **(C)**, and VEGF **(D)** in PBMC culture supernatants from surgical controls without visible endometriosis (−) and women with endometriosis (+). Violin plots display individual-value distributions, embedded boxplots show medians and interquartile ranges, and dots represent individual participants. Concentrations are expressed in pg/mL.

PHA stimulation induced marked changes in growth factor concentrations, but the magnitude of the response did not differ between women with endometriosis and controls (Table 4; Figure 2). In both groups, FGF and PDGF increased substantially after stimulation, and VEGF also increased with considerable inter-individual variability. In contrast, GM-CSF decreased after PHA stimulation in both groups (Table 4). After correction for multiple testing, none of the percent changes differed significantly between groups (all p-adj ≥ 0.72). Taken together, no between-group differences were observed in the percent changes after stimulation.

**Table 4:** Characteristic of each cytokine’s changes after PHA stimulation.

Characteristic	Surgical controls without endometriosis (−), *N* = 44	Endometriosis (+), *N* = 36	*p*-value	p-adj^1^
FGF change, %	227.0 (17.8, 370.5)	259.7 (62.5, 492.9)	0.38	0.72
GM-CSF change, %	−70.5 (−82.7, 98.9)	−48.6 (−87.9, 95.0)	0.74	0.74
PDGF change, %	189.4 (86.2, 391.1)	263.0 (118.0, 569.5)	0.37	0.72
VEGF change, %	88.1 (20.8, 284.4)	57.3 (2.3, 181.3)	0.54	0.72

Values are presented as median percent change (Q1, Q3). ^1^ Adjusted p-values were calculated using the Benjamini–Hochberg procedure. Change was calculated as: 100 × (PHA-stimulated concentration − baseline concentration)/baseline concentration. Patients with endometriosis are marked as (+), and surgical controls without endometriosis as (−).

Baseline PBMC secretion of FGF, PDGF, VEGF, and GM-CSF did not differ significantly between endometriosis cases and surgical controls after FDR correction. Descriptively, median FGF, PDGF, and VEGF values were higher after PHA stimulation, whereas GM-CSF values were lower; however, neither stimulated concentrations nor stimulation-induced changes differed significantly between groups after correction for multiple testing.

### 3.4 Subgroup analysis by endometriosis stage

Within the endometriosis group, 19 patients had rASRM stage I–II and 17 had stage III–IV disease. Compared with stage I–II, patients with stage III–IV disease were older (median 41.0 vs. 33.0 years; *p* = 0.035) and had a higher BMI (median 26.9 vs. 21.7 kg/m^2^; *p* = 0.04), while pregnancy history and menstrual cycle regularity were similar (Table 5). Because age and BMI differed between the rASRM I–II and rASRM III–IV subgroups, stage-stratified findings should be interpreted as exploratory and potentially confounded.

**Table 5:** Characteristic of two subgroups: ASRM I-II and III-IV.

Characteristic	rASRM I–II, *N* = 19	rASRM III–IV, *N* = 17	*p*-value
Age, years	33.0 (30.0, 39.0)	41.0 (37.0, 44.0)	**0.035**
Body mass, kg	62.0 (53.0, 80.0)	75.0 (58.0, 80.0)	0.23
Height, cm	166.0 (162.0, 169.0)	165.0 (162.0, 167.0)	0.99
BMI, kg/m^2^	21.7 (19.5, 27.4)	26.9 (22.8, 30.5)	**0.04**
Pregnancies, *n* (%)			0.37
0	6 (31.6%)	10 (58.8%)	
1	8 (42.1%)	3 (17.6%)	
2	3 (15.8%)	3 (17.6%)	
3	2 (10.5%)	1 (5.9%)	
Menstrual bleeding pattern, *n* (%)			0.72
Irregular	8 (47.1%)	8 (53.3%)	
Regular	9 (52.9%)	7 (46.7%)	

Values are presented as median (Q1, Q3) for continuous variables and *n* (%) for categorical variables. Bold *p*-values indicate nominal statistical significance at *p* < 0.05. Percentages may not sum to 100.0% because of rounding.

#### 3.4.1 Baseline growth factor levels by disease stage

Baseline growth factor concentrations were broadly comparable between stages (Table 6). Baseline PDGF was lower in stage III–IV than in stage I–II with a nominal *p*-value (*p* = 0.036), but this difference did not remain significant after FDR correction, (*p*-adj = 0.14). Baseline FGF, GM-CSF, and VEGF did not differ between stages after correction (Table 6).

**Table 6:** Characteristic of two subgroups: ASRM I-II and III-IV before PHA stimulation.

Characteristic	rASRM I–II, N = 19	rASRM III–IV, N = 17	p-value	p-adj^1^
FGF 0*, pg./mL	69.9 (52.7, 96.1)	69.9 (52.7, 83.9)	0.72	0.96
GM-CSF 0*, pg./mL	35.3 (5.2, 69.6)	8.4 (4.4, 92.3)	0.99	0.99
PDGF 0*, pg./mL	1,017.0 (329.0, 1,251.5)	499.0 (398.5, 693.8)	**0.036**	0.14
VEGF 0*, pg./mL	763.0 (397.9, 902.0)	598.8 (393.4, 809.1)	0.18	0.36

Values are presented as median (Q1, Q3). ^1^ Adjusted *p*-values were calculated using the Benjamini–Hochberg procedure. 0* — before PHA stimulation. Bold *p*-values indicate nominal *p* < 0.05; no comparison remained significant after FDR correction.

#### 3.4.2 Growth factor response to PHA stimulation by disease stage

PHA-induced percent changes did not differ significantly between stage subgroups after correction (Table 7). However, stage III–IV patients showed nominal trends toward a greater PDGF response (*p* = 0.09; *p*-adj = 0.18) and VEGF response (*p* = 0.08; *p*-adj = 0.18) compared with stage I–II (Table 7). These trends should be interpreted cautiously given the sample size and multiple testing. Although exploratory and not statistically significant after correction, these trends suggest a potentially greater stimulation-induced PDGF and VEGF release in advanced-stage disease, which is considered further in the Discussion.

**Table 7:** Characteristic of GF changes two subgroups: ASRM I-II and III-IV after PHA stimulation.

Characteristic	rASRM I–II, *N* = 19	rASRM III–IV, *N* = 17	*p*-value	*p*-adj^1^
FGF change, %	193.2 (26.7, 547.5)	280.3 (74.4, 487.8)	0.98	0.98
GM-CSF change, %	−80.7 (−87.1, 84.9)	30.3 (−89.2, 126.8)	0.74	0.98
PDGF change, %	191.9 (45.6, 408.0)	359.5 (158.0, 747.0)	0.09	0.18
VEGF change, %	31.9 (−7.4, 121.4)	86.8 (21.1, 267.4)	0.08	0.18

Values are presented as median percent change (Q1, Q3). ^1^ Adjusted *p*-values were calculated using the Benjamini–Hochberg procedure. Change was calculated as: 100 × (PHA-stimulated concentration − baseline concentration)/baseline concentration. No comparison remained significant after FDR correction.

### 3.5 Univariate logistic regression analysis

Univariate logistic regression was performed to assess associations between baseline clinical variables and baseline growth factor concentrations with endometriosis status (Table 8). No variable was significantly associated with endometriosis after FDR correction, including age, BMI, menstrual bleeding pattern, number of pregnancies, and baseline concentrations of FGF, GM-CSF, PDGF, and VEGF.

**Table 8:** Univariate logistic regression analysis for endometriosis status.

Characteristic	OR	95% CI	*p*-value	*p*-adj^1^
Age	1.02	0.96, 1.08	0.57	0.65
BMI	1.04	0.96, 1.13	0.37	0.65
Menstrual bleeding pattern				
Irregular	Ref.	—	—	—
Regular	1.33	0.53, 3.39	0.54	0.65
Pregnancies	0.77	0.48, 1.19	0.24	0.65
FGF 0*	1.00	0.99, 1.00	0.11	0.65
GM-CSF 0*	1.00	0.99, 1.01	0.71	0.71
PDGF 0*	1.00	1.00, 1.00	0.27	0.65
VEGF 0*	1.00	1.00, 1.00	0.53	0.65

^1^Adjusted *p*-values were calculated using the Benjamini–Hochberg procedure. 0* — before PHA stimulation. OR, odds ratio; CI, confidence interval; Ref., reference category.

Overall, neither baseline clinical variables nor baseline PBMC-derived growth factor concentrations were significantly associated with endometriosis status after correction for multiple testing.

## 4 Discussion

Our findings show that, under standardized 24-h *in vitro* culture conditions, PBMCs from women with endometriosis do not exhibit a generalized increase in FGF, PDGF, VEGF or GM-CSF secretion compared with PBMCs from surgical controls, either at baseline or after PHA stimulation. This argues against the notion that circulating mononuclear cells are intrinsically primed toward a hyper-angiogenic phenotype in endometriosis and instead supports a model in which key pro-angiogenic signals arise predominantly from local peritoneal and lesion-resident cells. Within the endometriosis group, exploratory stage-stratified analyses suggested nominal differences in PDGF and VEGF responses, but these did not withstand correction for multiple testing and should therefore be interpreted cautiously (11, 22). This interpretation is further supported by previous studies showing VEGF production by peritoneal-fluid macrophages, involvement of myeloid-derived suppressor cells in endometriosis progression, neutrophil-related G-CSF/IL-6-driven angiogenic signaling, and altered follicular-fluid cytokine profiles in women with endometriosis (27–30).

The lack of significant differences in FGF, VEGF and GM-CSF secretion by patients’ PBMCs indicates that our initial hypothesis of broadly elevated circulating immune cell-derived growth factors was not supported. This interpretation is reinforced by the literature: endometriotic lesions are known to harbor abundant macrophages, neutrophils and mast cells, which are considered primary sources of many of these mediators. For example, peritoneal macrophages in endometriosis patients produce high levels of VEGF and basic FGF (8), contributing substantially to the pro-angiogenic milieu. McLaren et al. demonstrated that peritoneal fluid macrophages are a major source of VEGF in endometriosis (9). It is therefore not surprising that peripheral immune cell cultures did not yield elevated VEGF — the bulk of VEGF in lesions likely originates from macrophages and endometrial stromal cells rather than from circulating lymphocytes and monocytes. Similarly, although GM-CSF has been reported as elevated in peritoneal fluid or serum in some studies, our data showed no increase in PBMC-derived GM-CSF, suggesting that other local cell populations account for any *in vitro* elevation. Indeed, reports on systemic cytokine levels in endometriosis are inconsistent: Malutan et al. found no significant elevation of bFGF, EGF or GM-CSF in the serum of women with advanced endometriosis and paradoxically observed lower circulating VEGF compared to controls, postulating that VEGF may be sequestered at lesion sites or that anti-angiogenic isoforms could mask systemic increases (13). Our findings mirror this discrepancy and reinforce the concept that pro-angiogenic activity in endometriosis is largely a localized phenomenon not reflected by peripheral immune cell output. In the endometriotic peritoneal niche, macrophages and neutrophils are key effector cells driving angiogenesis through IL-6, IL-8, TNF-*α*, VEGF and other mediators (22), while the role of circulating immune cells may be more permissive — creating conditions for those effector cells to act.

From a contextual standpoint, the literature suggests that regulatory T cells (Tregs) and other immunosuppressive populations contribute to the endometriotic milieu primarily through immunomodulation rather than direct angiogenic factor production. Zhou et al. highlighted IL10 and TGF-*β* as pivotal mediators through which regulatory immune populations help lesions evade immune attack (20). Although our study did not isolate or characterize Tregs specifically, the absence of elevated PBMC-derived VEGF, FGF or GM-CSF is consistent with a model in which immunoregulatory cells act as “enablers” rather than “effectors” — for instance, by suppressing natural killer (NK) and cytotoxic T-cell activity and by inducing macrophages toward a pro-angiogenic, pro-fibrotic M2 phenotype via IL-10 and TGF-β signaling (6). This interpretation aligns with emerging perspectives that endometriosis involves global immune dysregulation encompassing multiple cell types and complex interactions (6, 21). Our data, showing minimal differences in PBMC-secreted growth factors, are compatible with the view that no single immune cell subset single-handedly drives the cytokine network in endometriosis; rather, it is the crosstalk among diverse immune populations — including macrophages, neutrophils, Th17 cells and regulatory lymphocytes — that sustains the chronic inflammatory and pro-angiogenic environment (6, 21).

In published work on Tregs specifically, increased regulatory T-cell populations have been frequently reported in the peritoneal fluid and lesions of endometriosis patients (18, 23, 24). Khan et al. observed an accumulation of FOXP3^+^ Tregs in advanced-stage lesions (24), and endometriotic lesions have been shown to secrete chemokines such as CCL17, CCL22 and CCL20 to recruit immunoregulatory populations to the lesion site (18, 19). High levels of CCL17 and CCL22 attract Tregs and enhance their immunosuppressive function, which may indirectly support angiogenesis in implants (19). Paradoxically, a mouse study found that depletion of Tregs heightened inflammation and angiogenesis, ultimately accelerating lesion growth (6), highlighting the need for a balanced immune response. It must be emphasized that our study did not isolate Tregs from PBMCs and therefore cannot draw conclusions about Treg-specific function. The literature-based observations above are provided for interpretive context only. The absence of elevated PBMC-derived growth factors in our data is broadly consistent with a model in which angiogenic mediators are generated primarily within the local lesion niche, while immunoregulatory populations provide a permissive context for lesion persistence (3, 8).

Our findings help place emerging immunomodulatory strategies in context, although our study did not test any therapeutic intervention. Interest in targeting immune tolerance in endometriosis derives from the concept that relieving immunosuppression could improve clearance of ectopic endometrium. In a mouse model, Liu et al. reported smaller lesions after PLGA-encapsulated anti-CTLA-4 treatment aimed at modulating immune checkpoint activity (25).

Given that our PBMC cultures did not show increased secretion of VEGF, FGF or GM-CSF, any benefit of checkpoint-directed approaches — if confirmed — would more likely reflect altered cytotoxic activity and immune surveillance rather than reduced PBMC-derived angiogenic factor release. Checkpoint modulation in endometriosis remains investigational, and systemic immune-related adverse events are a key concern. Lesion-level recruitment pathways have also been proposed as targets: chemokines such as CCL17, CCL22 and CCL20 may contribute to immunoregulatory cell accumulation at lesion sites, and blockade of CCR4/CCR6 signaling has been hypothesized to shift the local immune balance (18, 19). These treatment implications are hypothesis-generating and require dedicated preclinical and clinical evaluation.

Beyond immune checkpoint concepts, therapeutic rationale often centers on mediators within the lesion and peritoneal compartment. Anti-angiogenic approaches (e.g., VEGF neutralization with bevacizumab or VEGFR inhibitors such as sorafenib and sunitinib) reduce lesion size and vascularization in animal models (8). However, translation to clinical care has been limited by safety and fertility considerations, and clinical evidence remains incomplete (8). Dopamine receptor agonists have also been explored as anti-angiogenic strategies in endometriosis, partly through modulation of VEGF-related pathways, although their clinical role remains investigational. Cytokine blockade has been explored with variable outcomes; for example, TNF-*α* inhibitors have shown mixed results in small clinical studies (5). Although we did not measure TNF-α directly, the inflammatory lesion milieu provides a rationale for testing such strategies (4, 6). GM-CSF has also been discussed as a candidate target because it can expand immunosuppressive myeloid populations that support lesions (6). In a mouse model, Zhang et al. reported that inhibiting the CXCL1/2–CXCR2 axis reduced myeloid recruitment and angiogenesis, leading to smaller lesions (26).

### 4.1 Limitations

This study has several limitations that should be considered when interpreting the findings. First, the sample size was modest (36 women with endometriosis and 44 controls), which, together with the need to correct for multiple comparisons, means that the study was primarily powered to detect relatively large between-group differences in growth factor secretion. Smaller but potentially biologically relevant differences may therefore have gone undetected, and the non-significant findings should not be over-interpreted as evidence of complete equivalence between groups.

Second, the 24-h culture period captures short-term ex vivo secretory responses and does not reproduce the chronic inflammatory and hormonal exposure characteristic of endometriosis *in vivo*. Longer culture periods, repeated stimulation models, co-culture systems, or parallel analysis of lesion and peritoneal samples may reveal disease-related differences not detectable in this short-term PBMC assay.

Third, we focused on a restricted panel of four growth factors (FGF, PDGF, VEGF and GM-CSF). Although these mediators are central to angiogenesis and myeloid cell regulation in endometriosis, they represent only a subset of the complex cytokine and chemokine network that shapes the endometriotic microenvironment. It remains possible that other soluble factors produced by PBMCs, including key inflammatory cytokines (e.g., IL-6, IL-8, TNF-*α*) or additional angiogenic mediators, show more pronounced alterations than those captured in the present analysis.

Fourth, all functional assays were performed on unfractionated PBMCs rather than on purified immune cell subsets. As a result, the measured concentrations reflect the integrated secretory output of a mixed population comprising T cells, B cells, NK cells and monocytes, and cannot be attributed to specific subsets such as regulatory T cells or particular monocyte or NK-cell populations. We also did not perform detailed immunophenotyping (e.g., flow cytometry with activation/exhaustion markers or FOXP3-defined Tregs), which limits our ability to relate secretory patterns to underlying changes in immune composition or activation states.

Fifth, although blood sampling was restricted to the early–mid follicular phase whenever possible (cycle days 5–12 based on menstrual calendar), we did not measure serum estradiol or progesterone. Calendar-based timing reduces, but does not eliminate, inter-individual variability in hormonal milieu, especially in women with irregular cycles, and residual endocrine differences may have influenced PBMC function. Finally, this was a single-center study conducted in a specific surgical population, which may limit the generalizability of the results to other settings or more diverse patient groups.

An important limitation is that the control group consisted of laparoscopic surgical controls rather than healthy asymptomatic volunteers. Although no visible endometriosis was detected intraoperatively, these women underwent surgery for infertility and/or pelvic pain, and alternative gynecological conditions may have been present. Therefore, the findings should be interpreted as comparisons with surgical controls without visible endometriosis, not with a disease-free healthy population.

Taken together, these limitations indicate that our data should be viewed as providing an initial, targeted assessment of PBMC-derived FGF, PDGF, VEGF and GM-CSF secretion in endometriosis, rather than a definitive characterization of systemic immune dysregulation. Future studies with larger, multicentre cohorts, broader cytokine panels, detailed immune phenotyping and subset-specific functional assays will be needed to build on and refine the conclusions presented here (7).

### 4.2 Conclusion

Under the standardized 24-h culture conditions used in this study, PBMCs from women with endometriosis did not show a generalized increase in secretory capacity for FGF, PDGF, VEGF or GM-CSF under basal conditions or after PHA stimulation. These findings do not support the concept of globally hypersecretory circulating PBMCs for the evaluated growth factors in endometriosis and are consistent with a model in which key pro-angiogenic and immunomodulatory signals are predominantly shaped within local peritoneal and lesion microenvironments. Given that PBMCs represent a mixed immune-cell population and measurements were performed at a single 24-h timepoint, future studies using purified cell subsets and parallel sampling of peripheral blood, peritoneal fluid and lesion tissue will be important to define compartment- and cell-type–specific contributions to endometriosis associated inflammation and angiogenesis.

## Data Availability

The raw data supporting the conclusions of this article will be made available by the authors, without undue reservation.
