# Supplementary material for: PBMC-derived FGF, PDGF, VEGF and GM-CSF secretion in endometriosis: a case–control in vitro study
Source: Front Med (Lausanne). 2026 Jun 4;13:1821695. doi: 10.3389/fmed.2026.1821695 (PMC13275388; doi:10.3389/fmed.2026.1821695)
Supplement: Supplementary file 1 [file Table_1.DOCX]

**Supplementary Tables**

Log2 fold-change analyses after PHA stimulation

**Supplementary Table S1. Log2 fold changes after PHA stimulation: surgical controls vs endometriosis.**

| **Characteristic** | **Surgical controls without endometriosis (-), N = 44** | **Endometriosis (+), N = 36** | **p-value** | **p-adj¹** |
| --- | --- | --- | --- | --- |
| FGF log2FC | 1.71 (0.24, 2.23) | 1.84 (0.69, 2.57) | 0.38 | 0.72 |
| GM-CSF log2FC | -1.77 (-2.54, 0.99) | -1.10 (-3.05, 0.96) | 0.74 | 0.74 |
| PDGF log2FC | 1.53 (0.90, 2.29) | 1.86 (1.12, 2.74) | 0.37 | 0.72 |
| VEGF log2FC | 0.91 (0.27, 1.94) | 0.65 (0.03, 1.48) | 0.54 | 0.72 |

Values are presented as median log2 fold change (Q1, Q3). Log2 fold change was calculated as log2(PHA-stimulated concentration / baseline concentration).

¹ Adjusted p-values were calculated using the Benjamini-Hochberg procedure. PHA, phytohemagglutinin; rASRM, revised American Society for Reproductive Medicine.

**Supplementary Table S2. Log2 fold changes after PHA stimulation according to endometriosis stage.**

| **Characteristic** | **rASRM I-II, N = 19** | **rASRM III-IV, N = 17** | **p-value** | **p-adj¹** |
| --- | --- | --- | --- | --- |
| FGF log2FC | 1.55 (0.61, 2.53) | 1.93 (0.80, 2.56) | 0.98 | 0.98 |
| GM-CSF log2FC | -2.37 (-2.85, 0.82) | 0.38 (-3.21, 1.18) | 0.74 | 0.98 |
| PDGF log2FC | 1.55 (0.59, 2.22) | 2.20 (1.37, 3.08) | 0.09 | 0.18 |
| VEGF log2FC | 0.40 (-0.09, 1.11) | 0.90 (0.28, 1.88) | 0.08 | 0.18 |

Values are presented as median log2 fold change (Q1, Q3). Log2 fold change was calculated as log2(PHA-stimulated concentration / baseline concentration).

¹ Adjusted p-values were calculated using the Benjamini-Hochberg procedure. PHA, phytohemagglutinin; rASRM, revised American Society for Reproductive Medicine.
